# Stability in the Face of Change: Lifelong Experience-Dependent Plasticity in the Sensory Cortex

**DOI:** 10.3389/fncel.2020.00076

**Published:** 2020-04-21

**Authors:** Adema Ribic

**Affiliations:** Department of Psychology, College and Graduate School of Arts and Sciences, University of Virginia, Charlottesville, VA, United States

**Keywords:** cortex, plasticity, interneuron, experience, synapse, cholinergic

## Abstract

Plasticity is a fundamental property of the nervous system that enables its adaptations to the ever-changing environment. Heightened plasticity typical for developing circuits facilitates their robust experience-dependent functional maturation. This plasticity wanes during adolescence to permit the stabilization of mature brain function, but abundant evidence supports that adult circuits exhibit both transient and long-term experience-induced plasticity. Cortical plasticity has been extensively studied throughout the life span in sensory systems and the main distinction between development and adulthood arising from these studies is the concept that passive exposure to relevant information is sufficient to drive robust plasticity early in life, while higher-order attentional mechanisms are necessary to drive plastic changes in adults. Recent work in the primary visual and auditory cortices began to define the circuit mechanisms that govern these processes and enable continuous adaptation to the environment, with transient circuit disinhibition emerging as a common prerequisite for both developmental and adult plasticity. Drawing from studies in visual and auditory systems, this review article summarizes recent reports on the circuit and cellular mechanisms of experience-driven plasticity in the developing and adult brains and emphasizes the similarities and differences between them. The benefits of distinct plasticity mechanisms used at different ages are discussed in the context of sensory learning, as well as their relationship to maladaptive plasticity and neurodevelopmental brain disorders. Knowledge gaps and avenues for future work are highlighted, and these will hopefully motivate future research in these areas, particularly those about the learning of complex skills during development.

## Introduction

The adaptability of neural circuits to different contexts is central to their function. The heterogeneity and unpredictability of our experiences dictate multiple forms of this plasticity, from rapid adaptations to unfamiliar environments to gradual acquisition of skills. It is widely accepted that plasticity is a characteristic of the young brain, enabling early experiences to exert tremendous influence over the development of most, if not all skills and abilities. Seminal studies by Lorenz (1935) almost a century ago were the first to formally describe a brief window of time during which newly-hatched birds become attached to the first large moving object they encounter. He proposed that this “critical” period of sensitivity to the environment enables hatchlings to recognize their parents (Lorenz, 1935). Later studies demonstrated that adequate extrinsic (environmental) and intrinsic (genetic) factors during these periods guide the establishment of our basic senses: vision (Hubel and Wiesel, 1964), hearing (Knudsen et al., 1984), touch (Simons and Land, 1987), taste (Hill and Przekop, 1988; Mennella et al., 2011), and olfaction (Franks and Isaacson, 2005; Ma et al., 2014; Tsai and Barnea, 2014). Critical periods became defined as brief windows of time during which neural circuits are plastic and can be robustly and quickly shaped by their immediate environment to optimize their function in that environment. In almost all instances, plasticity during the critical periods is sufficiently high to allow circuits to be remodeled by passive exposure to the environmental stimuli. Higher cognitive functions have a period of sensitivity as well: socialization during adolescence can affect behavioral task performance (Einon and Morgan, 1977), and timely exposure to proper acoustic stimuli facilitates vocal learning (Immelmann, 1969; Eales, 1985).

Over time, research on critical period plasticity became synonymous with experience-dependent plasticity, especially in the primary visual cortex (V1). Classical studies by David Hubel and Thorsten Wiesel demonstrated that the closure of one eye for a brief period during early life induces plasticity of eye-specific cortical columns, where open eye columns expand at the expense of closed eye columns (Wiesel and Hubel, 1963; Hubel and Wiesel, 1964; Hubel et al., 1977). This robust reorganization of visual cortical areas, aptly named ocular dominance (OD) plasticity, was absent after eye closure in adults, leading to the dogma of aplastic mature brain (Hubel and Wiesel, 1970; LeVay et al., 1980). How are then adult brains responding to diverse experiences if lifelong learning requires plasticity?

More recent methodological advances in neuroscience enabled systematic re-examination of plasticity in adults and challenged the notion of an inflexible adult brain. Robust activity-driven changes in mature V1 circuits have been demonstrated during learning, as well as after monocular lid suture performed in adulthood (Ball and Sekuler, 1982; Dragoi et al., 2000; Yao and Dan, 2001; Sawtell et al., 2003; Sato and Stryker, 2008; Lunghi et al., 2011). However, in contrast to critical period plasticity that can be driven by passive exposure to the relevant information, adult plasticity usually requires attentiveness to the environment and top-down modulation by frontal brain regions (Seitz and Dinse, 2007; de Villers-Sidani et al., 2008).

Initial mechanistic studies of both adult and developmental plasticity described vital roles of excitatory neurotransmission mediated by N-methyl-D-aspartate (NMDA) receptors in the initiation and offset of plasticity (Daw et al., 1999; Feldman, 2003; Sawtell et al., 2003; Sato and Stryker, 2008; Cho et al., 2009). More recent studies have focused on elucidating cellular and circuit mechanisms that drive the plastic changes in the developing and adult brain, and these are the topic of this review. Studies of plasticity in the visual and auditory cortices cortex have been central to our current understanding of this process, and these are compared to draw a few general rules. Distinct circuit mechanisms of plasticity through the life span are discussed in the context of neurodevelopmental disorders, and circuit mechanisms employed both during development and in adulthood are highlighted to identify future directions of research that may be crucial for our understanding of lifelong learning.

## Plasticity in The Developing Cortex

Learning during postnatal development is thought to happen passively through exposure to the appropriate environment during the critical periods (Lorenz, 1935; Hensch, 2004; Hübener and Bonhoeffer, 2014). Heightened brain plasticity during this time enables robust and lasting adaptations to the immediate surroundings (Lorenz, 1935; Kreile et al., 2011; Zhou et al., 2011). Classical examples are found in sensory areas, where exposure to adequate sensory stimuli early in life leads to precise representations of the natural world (Lillard and Erisir, 2011). In the visual cortex (V1), essential features of visually responsive neurons such as their preference for the direction of moving stimuli, emerge without any visual experience (Hubel and Wiesel, 1963; Daw and Wyatt, 1976; Chapman and Stryker, 1993; Rochefort et al., 2011; Ko et al., 2013). However, gross vision is extremely sensitive to the balance and quality of visual inputs after the critical period for OD plasticity opens (Giffin and Mitchell, 1978; Harwerth et al., 1986; Prusky and Douglas, 2003). Even passive exposure to an environment dominated by a single feature (like oriented stripes) during the critical period is enough to shift the response preference of V1 neurons towards that feature (Daw and Wyatt, 1976; Kreile et al., 2011). Similarly, rearing young rats in an acoustic environment dominated by a single frequency accelerates the development of areas representing that frequency in the auditory cortex (A1) and expands their cortical representation (Zhang et al., 2001).

In both V1 and A1, the development of inhibitory γ-aminobutyric acid (GABA)-releasing circuitry seems to be a prerequisite for the critical period to open (Hensch et al., 1998; Takesian et al., 2013). This may be the case in other cortical areas as well, since sensory activity strongly drives the development of cortical inhibition (Chittajallu and Isaac, 2010). Studies suggest that a gradual rise in the activity of GABAergic neurons after the eye-opening (postnatal day 14 in mice) initiates the critical period for visual plasticity (Morales et al., 2002; Kuhlman et al., 2010; Lazarus and Huang, 2011). Deletion of synapse-localized GABA-synthetizing enzyme glutamic acid decarboxylase 65 (GAD65) blocks the critical period onset in the V1, while an intracortical infusion of GABA-receptor agonists immediately after eye-opening initiates a premature critical period (Hensch et al., 1998; Fagiolini and Hensch, 2000). It is still unclear which inhibitory neuron subtypes permit the critical period to open, but mutations in alpha-1 GABA receptor that is preferentially expressed in Parvalbumin^+^ (PV) basket interneurons block the effects of GABA-receptor agonists in precritical period mice (Fagiolini et al., 2004). In line with this, loss of local excitation onto PV cells in V1 precludes critical period onset (Gu et al., 2013) and genetic mouse models of premature PV maturation display premature critical period opening and impaired maturation of binocular vision (Huang et al., 2015; Krishnan et al., 2015).

Inhibitory circuits are particularly sensitive to sensory input during the critical period. In the V1, sensory deprivation by dark rearing of mice from birth prevents the developmental increase of GABAergic neurotransmission and keeps the cortex sensitive to monocular deprivation well into adulthood (Fagiolini et al., 1994; Morales et al., 2002), and in the primary auditory cortex (A1) hearing loss retards the maturation of cortical inhibitory tone (Kotak et al., 2008; Xu et al., 2010). Experience-dependent maturation of cortical inhibition continues through the critical period and recent studies suggest that the level of inhibitory tone must reach a certain level not just for critical period opening, but for the closure as well. Chemogenetic and genetic suppression of PV interneuron activity in the V1 before the critical period closes can extend plasticity into adulthood (Kuhlman et al., 2013; Ribic et al., 2019), indicating that disinhibition is permissive for plasticity *after* the first inhibitory threshold is crossed and the critical period has begun (Ma et al., 2013). In agreement, disinhibition occurs rapidly after sensory deprivation during the critical period (Gambino and Holtmaat, 2012; Kuhlman et al., 2013; Takesian et al., 2013; Gainey and Feldman, 2017; Miska et al., 2018). However, lasting changes in the dynamics of inhibitory synapses after sensory deprivation may be circuit dependent. In the A1, developmental hearing loss can induce enduring disinhibition and behavioral deficits that can be improved by restoring the inhibitory tone (Mowery et al., 2019), whereas visual deprivation triggers circuit-specific changes in the V1 that can result in increased inhibitory tone in local cortical circuits (Maffei et al., 2006; Kannan et al., 2016; Miska et al., 2018). While it is still unclear if these differences stem from distinct mechanisms of homeostasis in response to sensory manipulations, studies agree that the fast dynamics of inhibitory neurotransmission may be the key to sensory adaptations during the critical periods (Gainey and Feldman, 2017). Intriguingly, transplantation of cortical embryonic interneurons into adult V1 triggers another window of visual plasticity identical to the juvenile critical period in the onset and duration (Southwell et al., 2010; Davis et al., 2015). The host cortex becomes responsive to monocular deprivation once transplanted interneurons reach the critical period age, indicating that additional intrinsic regulators of interneuron maturation and critical period timing exist. More recent work indicates that the host’s response to the transplantation process itself is essential for the maintenance of plasticity after monocular deprivation (Hoseini et al., 2019). It is currently unknown what exactly elicits the new plasticity in the host cortex, transient disinhibition or circuit destabilization, as well as what factors intrinsic to both host and transplanted cells may regulate this process.

**Figure 1:**
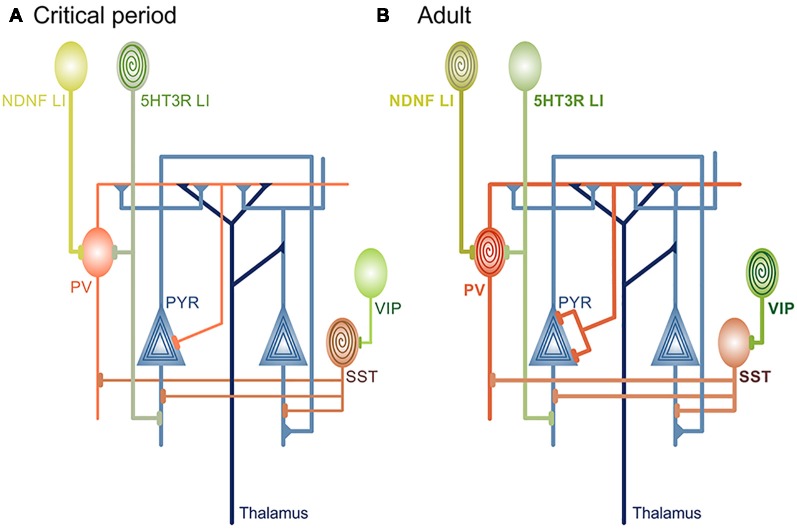
Circuit changes during cortical maturation. **(A)** Rising levels of inhibitory neurotransmission are a prerequisite for onset of developmental, “passive” learning phase that occurs during the critical periods. Different types of interneurons are the source of inhibition during cortical maturation: soma-targeting Parvalbumin (PV), dendrite-targeting Somatostatin (Sst) and 5HT3-receptor^+^ layer I (LI) interneurons (also dendrite-targeting). It is currently unknown if and how disinhibitory vasoactive intestinal peptide (VIP) interneurons contribute to critical period plasticity. LI and Sst interneurons are sensitive to neuromodulatory inputs (spiral) before the critical period closes and robustly depolarize in response to acetylcholine (ACh) agonists or stimulation of basal forebrain. Behavioral significance of this sensitivity is yet to be defined. **(B)** As the cortex matures, neuromodulation-induced depolarization of 5HT3 LI and Sst interneurons is attenuated, in contrast to soma-targeting PV that begin to rapidly increase firing rate in response to cholinergic agonists. At the same time, the level of cortical inhibitory neurotransmission reaches a level that is no longer permissive for rapid circuit changes induced by passive exposure to environmental stimuli. Disinhibitory ACh-sensitive VIP and neuron-derived neurotrophic factor (NDNF)^+^ LI interneurons become central for plasticity and learning through the regulation of Sst and PV activity, respectively. PYR neurons are sensitive to neuromodulatory inputs throughout the lifetime.

Excitatory inputs onto PV cells display experience-dependent and input-specific changes in strength and plasticity during development, providing the synaptic basis for critical period plasticity (Chittajallu and Isaac, 2010; Lazarus and Huang, 2011; Lu et al., 2014; Miao et al., 2016; Guan et al., 2017; Ferrer et al., 2018). In the V1, short-term plasticity of local cortical but not thalamic excitation onto PV cells is selectively regulated before and during the critical period (Lu et al., 2014; Miao et al., 2016; Figure 1A). In agreement, selective loss of local excitatory inputs onto PV interneurons prevents critical period opening (Gu et al., 2013). However, loss of thalamic inputs onto PV cells prevents critical period closure (Ribic et al., 2019), highlighting separate roles of different synapse types in cortical plasticity (Miska et al., 2018). Increased sensory-driven dynamics of local inputs onto PV interneurons before and during the critical period parallels the maturation of their output (Kuhlman et al., 2010; Lu et al., 2014). Such a relationship may facilitate the maturation of correlated activity between local networks of PV and pyramidal neurons, necessary for precise sensory processing (Kuhlman et al., 2011; Lu et al., 2014). Inputs from the sensory thalamus may confer sensitivity to the quality of input, timing the critical period closure to the maturation of sensory-evoked responses throughout the cortex (Toyoizumi et al., 2013; Gu and Cang, 2016; Shen and Colonnese, 2016; Ribic et al., 2019). Future studies can now address the roles of cortical feedback to the sensory thalamus in the coordination of activity between these two structures during the cortical maturation (Thompson et al., 2016).

PV interneurons are not the only inhibitory cell type whose maturation is sensitive to sensory input. Inhibition mediated by dendrite-targeting Somatostatin (Sst) interneurons in the V1 is also dynamic before critical period opening (Lazarus and Huang, 2011; Guan et al., 2017). Sst interneurons in the V1 are strongly depolarized by acetylcholine (ACh) agonists before critical period closure (Yaeger et al., 2019; Figure 1A). While this provides a link to the previously described restriction of critical period duration by neuromodulatory mechanisms (Morishita et al., 2010), it is still unclear if restricted sensitivity of Sst interneurons to cholinergic signaling contributes to critical period closure. Experiments using Sst-specific manipulation of ACh receptor expression would provide a likely answer. Similar to SSt interneurons, nicotine strongly depolarizes serotonin receptor 3 (5HT3) Layer I (LI) interneurons in the primary auditory cortex (A1) only early postnatally in mice (Takesian et al., 2013; Figure 1A). LI interneurons normally inhibit PV and pyramidal cells, and suppression of their activity blocks the initiation of the critical period for tonotopic map plasticity in the A1 (Takesian et al., 2013). As mentioned earlier, rearing mice exposed to a single sound frequency during the auditory critical period (P11-P15) changes the cortical representation of tones in the A1 and expands the areas that are responsive to the reared frequency (Zhang et al., 2001; Barkat et al., 2011). Preventing this process through the silencing of PV-inhibiting LI interneurons is at first glance contradictory to V1 plasticity, where an accelerated increase of PV-mediated inhibition can initiate a premature critical period (Huang et al., 2015). However, the bulk of PV maturation throughout the cortex occurs after P15 (Gao et al., 2000; Oswald and Reyes, 2008; Lazarus and Huang, 2011), indicating that LI interneurons are the main source of inhibition during the auditory critical period.

Experimental evidence hence supports that sensory, bottom-up pathways drive crossing of a circuit-specific inhibitory threshold and resulting changes in sensory-evoked cortical activity may serve as a signal for a critical period to open (Toyoizumi et al., 2013; Shen and Colonnese, 2016). Continued maturation of cortical inhibitory tone can contribute to critical period closure (Dorrn et al., 2010; Ribic et al., 2019), but the tapering of juvenile plasticity is additionally controlled by neuromodulation (Morishita et al., 2010; Blundon et al., 2017), NMDA receptor composition at the synapse (Cho et al., 2009), the developmental decline in the density of silent synapses (Huang et al., 2015; Sun et al., 2016) and different structural synaptic brakes on plasticity (Pizzorusso et al., 2002; McGee et al., 2005). Neuromodulators such as ACh and serotonin are widely accepted as key mechanisms that control arousal and attention (Zagha and McCormick, 2014; McCormick et al., 2015; McGinley et al., 2015; Thiele and Bellgrove, 2018). They also affect plasticity in developing cortical circuits, suggesting that some forms of juvenile plasticity can be dependent on behavioral states (Bear and Singer, 1986; Lillard and Erisir, 2011). Is learning and adaptation during critical periods truly passive as previously thought? While it would be highly advantageous that basic sensory functions do not require more than simple exposure to environmental stimuli for functional maturation, complex skills, cognitive functions and behaviors likely require the involvement of higher brain regions and/or of neuromodulatory systems (Puzerey et al., 2018; Nardou et al., 2019). Future studies are bound to address this in more detail, as well as whether coordination of thalamic and local circuits represents a way to time cortical critical period closure (Toyoizumi et al., 2013; Gu and Cang, 2016).

## Plasticity in The Adult Cortex

Stable circuitry is a defining characteristic of the mature brain. After the critical periods close, neural plasticity tapers off to ensure the preservation of function. Yet, studies in sensory cortices have demonstrated extensive cortical remodeling and plasticity in the adult brain during and after learning (Jenkins et al., 1990; Recanzone et al., 1993; Schoups et al., 2001), sensory conditioning (Galambos et al., 1956; Weinberger, 1993), localized neural stimulation (Frégnac et al., 1988, 1992; Debanne et al., 1998; Schuett et al., 2001) and loss of peripheral input (Merzenich et al., 1983; Pons et al., 1988; Robertson and Irvine, 1989; Kaas et al., 1990; Chino et al., 1992). While passive exposure to environmental information can in some cases drive plasticity in the mature brain (Cooke and Bear, 2010), most instances of learning-induced plasticity in adults usually require attention-driven enhancement of detection of and response to relevant information. This type of plasticity is robustly induced by learning a task through practice or conditioning and seems to have a role in the early stages of learning, but not in the maintenance of newly acquired skills (Molina-Luna et al., 2008; Yotsumoto et al., 2008; Reed et al., 2011). During perceptual learning, the subject trains to detect or discriminate a feature after repeated practice. In associative learning through conditioning, discrimination of the feature is paired and associated with either a reward or punishment. Both have been adapted for a wide variety of sensory tasks that result in robust cortical reorganization and best-known examples include studies of auditory learning.

Much like neurons in the V1 have a preference for orientation or direction of visual features in the environment, A1 neurons have a preferred tonal frequency that induces a maximal response in a given neuron. Further, A1 organization also follows the sensory topographic rules, where A1 neurons are grouped by their preferred frequency and arranged in a gradient that faithfully represents the auditory environment. As described in previous section, frequency maps in young animals show robust plasticity after passive exposure to sounds (Zhang et al., 2001; Barkat et al., 2011). Map plasticity in adults is usually not induced by passive exposure, but by learning.

The behavior has long been known to influence the firing of neurons in the A1 (Hubel et al., 1959), and a large body of work demonstrated how associative tasks where rewarding or noxious stimuli are paired with the presentation of a distinct tone can induce rapid and lasting adaptations in the tuning of neurons in the A1 (Beaton and Miller, 1975; Bakin and Weinberger, 1990; Edeline and Weinberger, 1993; Blake et al., 2002; Polley et al., 2004; Weinberger, 2004; Fritz et al., 2005; McGann, 2015). A1 neurons can increase their firing in response to the presentation of the conditioned tone (Beaton and Miller, 1975; Bakin and Weinberger, 1990), and representation of conditioned frequency in the cortex can in some cases expand (Polley et al., 2006). Similarly, discrimination tasks where animals are trained to discriminate an auditory frequency after repeated presentation of tones can modulate the firing of single neurons in the A1 during task performance (Miller et al., 1972) and enlarge the cortical representation of the trained frequency (Recanzone et al., 1993). Much like the plasticity in A1, tactile training enlarges the representation of the corresponding skin area in the somatosensory cortex (Recanzone et al., 1992). Visual training, on the other hand, does not modify the topography of V1, but the plasticity is evident in the visual responsiveness of V1 neurons (Schoups et al., 2001; Yang and Maunsell, 2004; Yan et al., 2014; Goltstein et al., 2018).

As mentioned, plastic changes during sensory learning tasks in adults often depend on the behavior. These changes occur selectively during task performance (Miller et al., 1972) and studies confirm that attentiveness to the task can induce rapid adaptations of both single neuron firing and maps in mammalian A1 (Grady et al., 1997; Fritz et al., 2003; Otazu et al., 2009; Lee and Middlebrooks, 2011; Niwa et al., 2012; Schreiner and Polley, 2014; Irvine, 2018). Further, in most instances of sensory task learning through repeated practice, attentiveness and task engagement enhance performance, indicating that higher-order frontal brain regions modulate learning and associated plasticity in adults (Ahissar and Hochstein, 1993; Seitz and Dinse, 2007; Lee and Middlebrooks, 2011; Mukai et al., 2011; Byers and Serences, 2012; Niwa et al., 2012; Caras and Sanes, 2017). Brain region commonly implicated in attention is the basal forebrain, consisting of several nuclei that send out cholinergic projections to all sensory areas and that undergoes plastic changes in response to learning paradigms (Guo et al., 2019). Sensory areas themselves show differential distribution of ACh receptors, indicating that sensory modalities can be distinctly modulated by top-down mechanisms (Levey et al., 1991). Pairing stimulation of the nucleus basalis in the basal forebrain with the presentation of tones induces rapid plasticity in the A1 (Bakin and Weinberger, 1996; Kilgard and Merzenich, 1998; Froemke et al., 2007; Puckett et al., 2007; Guo et al., 2019) and V1 (Goard and Dan, 2009; Bhattacharyya et al., 2013; Kang et al., 2014), while pairing it with auditory or visual discrimination tasks enhances performance in those tasks (Froemke et al., 2013; Pinto et al., 2013). As already stated, learning-induced plasticity appears to support task learning, but not the maintenance of newly acquired abilities (Reed et al., 2011), and the question remains whether continued plasticity would improve or deteriorate the consolidation of learned tasks. Also, and given that the high level of inhibition restricts plasticity after the critical periods close, how are these plastic changes during learning initiated?

In the V1, cholinergic inputs from the basal forebrain can activate disinhibitory Vasoactive Intestinal Peptide (VIP) interneurons to enhance visual plasticity in adults (Fu et al., 2014, 2015; Figure 1B). In the A1, conditioning through pairing auditory cues with noxious stimuli acts through activation of disinhibitory Neuron-Derived Neurotrophic Factor (NDNF)^+^ Layer I interneurons by inputs from the nucleus basalis (Letzkus et al., 2011; Abs et al., 2018; Figure 1B). Finally, pairing the stimulation of nucleus basalis with the presentation of tones induces enhancement of excitatory postsynaptic currents (EPSCs) in the A1, but this is preceded by a rapid reduction in inhibitory postsynaptic currents (IPSCs; Froemke et al., 2007). Disinhibition, therefore, appears to be a requirement for initiation of plasticity in adult sensory cortices, akin to plasticity during the critical periods (Letzkus et al., 2011; Kuhlman et al., 2013). In agreement, reducing gross inhibition in the adult A1 and V1 either pharmacologically or through other manipulations restores robust sensitivity to sensory manipulations (Pizzorusso et al., 2002; Maya Vetencourt et al., 2008; Harauzov et al., 2010; Cisneros-Franco and de Villers-Sidani, 2019), but interestingly, reduction of inhibition seems dispensable for the maintenance of plasticity after it has been initiated (Kaplan et al., 2016). On a network level, learning-induced plasticity promotes the correlated activity of PYR and PV neurons and formation of their ensembles (Khan et al., 2018), but it is unknown whether transient disinhibition precedes this process, as well as if these network changes depend on cholinergic inputs originating in the forebrain. Studies suggest a sequence of events during adult sensory learning, where attentiveness to a task causes circuit disinhibition modulated by top-down inputs from the frontal brain regions. This disinhibition is transient (Froemke et al., 2007) and sufficient to induce rapid plasticity of sensory maps and single neuronal responses. Plasticity seems to support the formation of task-relevant neuronal ensembles and learning in the early stages but, much like disinhibition, it is transient and not necessary for the maintenance and stabilization of learned skills (Reed et al., 2011; Khan et al., 2018). New circuit approaches capable of cell- or input-specific manipulation will be crucial in determining whether this is indeed the case.

**Figure 2:**
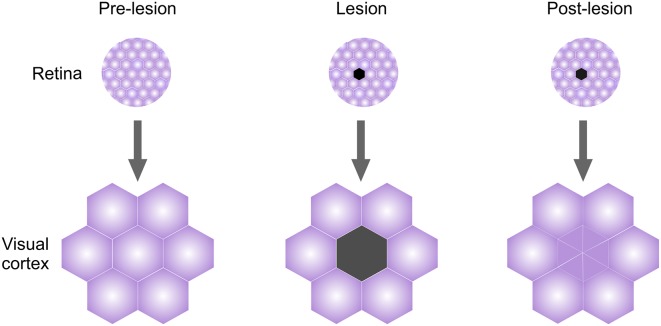
Lesion projection zone (LPZ) plasticity in adult visual cortex (V1). Retinal visual field organization is mapped faithfully onto the V1. Lesioning an area in the retina will initially cause loss of responses in the corresponding cortical region called LPZ. However, even while retina does not recover, LPZ neurons recover visual responses over time and acquire visual properties of neighboring visual fields.

Another form of plasticity that is extensively studied in the adult cortex is plasticity induced by sensory deafferentation. Injuries to sensory inputs result in robust cortical remodeling that occurs on timescales slower than those observed during learning (Sammons and Keck, 2015). Among the best-known examples is deafferentiation in the V1, where focal retinal lesions initially induce loss of visual responses in the corresponding cortical lesion projection zone (LPZ). LPZ neurons, however, recover their responses over time (Kaas et al., 1990; Gilbert and Wiesel, 1992; Abe et al., 2015; Figure 2). These responses do not reflect the original, pre-lesion organization of the V1; instead, the recovered responses reflect the activity of neighboring intact visual fields (Kaas et al., 1990; Gilbert and Wiesel, 1992; Keck et al., 2008; Figure 2). Both functional and structural remodeling of local circuits mediate LPZ plasticity and, similar to deprivation-induced plasticity in the V1, inhibitory circuits reorganize ahead of excitatory circuits (Chen et al., 2011a,b; Keck et al., 2011; Kuhlman et al., 2013). A subset of cortical interneurons positive for Neuropeptide Y (NPY) shows fewer excitatory inputs and axonal boutons immediately post-lesioning, with the extent of changes decreasing towards the area neighboring the LPZ (Keck et al., 2011). Dendritic trees of Parvalbumin and Calretinin positive neurons remodel as well in the days following the lesion, indicating global reorganization of inhibitory circuitry in the LPZ and the area surrounding it (Marik et al., 2014). Pyramidal (PYR) neurons, in contrast, show no changes in the overall density of excitatory inputs after the lesioning due to an increase in the overall turnover of dendritic spines (Keck et al., 2008, 2011). However, the fraction of persistent newly formed spines on LPZ PYR neurons increases over time, paralleling the functional recovery of these neurons (Keck et al., 2008).

Local connectivity in the area surrounding the LPZ reorganizes as well, providing a structural basis for the observed functional changes in visual field remapping. Axons of pyramidal neurons in the peri-LPZ zone initially sprout and grow into the LPZ, followed by a period of high turnover, in a process comparable to developmental growth and refinement of axonal arbors (Darian-Smith and Gilbert, 1994; Yamahachi et al., 2009). A similar process happens after whisker plucking in the rodent somatosensory cortex, where axons of PYR cells from regions responding to intact whiskers outgrow into the deprived regions (Marik et al., 2010). Interestingly, axons of inhibitory neurons within the deprived area outgrow towards the spared regions, suggesting that there are structural mechanisms in place to enable the homeostasis of excitatory-inhibitory network balance (Marik et al., 2010; Nahmani and Turrigiano, 2014). In the A1, auditory neurons increase their firing rates after cochlear denervation which partially restores sensitivity to tones (Chambers et al., 2016). This process is likely mediated by local disinhibition of PV interneurons (Resnik and Polley, 2017) and rapid plasticity of inhibitory neurons within the deprived area may serve to facilitate the plasticity both within and outside the deprived regions (Feldman, 2009; Chen et al., 2011a; Li et al., 2014; Gainey and Feldman, 2017).

Structural changes in inhibitory neurons seen in LPZ suggest that dynamic changes in inhibitory tone may represent a global rule for initiation of plasticity and adjustment of cortical activity following peripheral injury in adults (Keck et al., 2011; Resnik and Polley, 2017), but it is unclear if this plasticity can be modulated by top-down pathways. Identifying precise cellular mechanisms of plasticity after injury, as well as defining potential roles of attention in this process may prove to be crucial for development of novel translational approaches for treatment of brain injury (Voss et al., 2017).

## Regulation of Plasticity: Timing Is Everything

Ever since the functional [inhibition (Hensch et al., 1998)] and structural [ECM and myelin (Pizzorusso et al., 2002; McGee et al., 2005)] brakes on juvenile plasticity in the V1 have been described, a growing number of studies reported ways to restore critical period-like plasticity in the adult brain (Maya Vetencourt et al., 2008; Bavelier et al., 2010; Carulli et al., 2010; Harauzov et al., 2010; Morishita et al., 2010; Zhou et al., 2011; Beurdeley et al., 2012; Hensch and Bilimoria, 2012; Gervain et al., 2013; Davis et al., 2015). While the limited and transient restoration of developmental plasticity in the mature cortex may be beneficial for reestablishing the correct circuit architecture after brain injury and in numerous disorders characterized by aberrant connectivity (Levelt and Hübener, 2012; Hübener and Bonhoeffer, 2014; Ribic and Biederer, 2019), prolonging critical periods into adulthood is detrimental. Mice lacking structural brakes on plasticity, such as ECM, myelin-associated receptor NogoR, the cell adhesion molecule 1, as well as adult mice transplanted with embryonic interneurons, show deprivation-induced plasticity well into adulthood, but also display impairments in associative conditioning learning paradigms (Gogolla et al., 2009; Akbik et al., 2013; Park et al., 2016; Yang et al., 2016; Banerjee et al., 2017; Thompson et al., 2018). This indicates that the tapering of developmental plasticity is a requirement for the transition to adult forms of learning. However, circuit mechanisms of this transition are unclear. Differential sensitivity of interneuron subtypes to cholinergic modulation before and after the critical period onset would enable a simple way to fine-tune cortical activity and promote the switch from passive to active learning and from glutamatergic bottom-up to neuromodulatory top-down pathways (Takesian et al., 2013; Yaeger et al., 2019). Interestingly, genetically attenuating cholinergic signaling throughout the brain can extend critical period into adulthood and even enhance some forms of associative learning in mice (Miwa et al., 2006; Morishita et al., 2010). Detailed, cell-type-specific dissection of roles that neuromodulatory control of inhibition plays in developmental and adult learning, as well as in the switch from one type to another, will be crucial to consolidate these with other findings on cholinergic modulation of associative learning.

Offset of developmental plasticity may be critical for learning in adults, but the precise onset of developmental plasticity seems to be vital for passive forms of learning in children. Precocious opening of a visual critical period leads to impaired processing of visual inputs and has been observed in mouse models of Rett syndrome (Wang et al., 2013; Krishnan et al., 2015). Mouse models of other neurodevelopmental disorders, including autism spectrum disorder (ASD) and schizophrenia, frequently display abnormal critical period timing and a myriad of sensory impairments, highlighting the significance of accurately timed plasticity for learning during development (Mei and Xiong, 2008; Durand et al., 2012; Krishnan et al., 2015; Gu et al., 2016; Sun et al., 2016). However, a clear picture of circuit mechanisms that go awry in these disorders is missing. Balance of excitatory and inhibitory neurotransmission (E/I) that arises during the critical periods has been proposed as the cause of major cognitive and behavioral symptoms of ASD (Rubenstein and Merzenich, 2003; Toyoizumi et al., 2013; Nelson and Valakh, 2015). Recent systematic examination of sensory circuit properties in different ASD mouse models suggests that the E/I imbalance is a homeostatic compensatory response to decreased local cortical inhibition, but it is unclear how the deficit in inhibition arises in the first place (Antoine et al., 2019). Co-morbidity of ASD and attention disorders implicates neuromodulatory systems in the etiology of ASD, and recent studies confirm that cholinergic regulation of inhibitory cortical networks can regulate critical period timing (Takesian et al., 2013; Yaeger et al., 2019). Systematic examination of properties of different neuromodulatory circuits in ASD mouse models is currently missing, both in developing and adult brain. Potential future discoveries in this area might fill the missing gaps in understanding how the transition from developmental to adult forms of learning occurs on cellular and circuit levels.

## Continuity of Plasticity During Cortical Maturation

Recent research provided abundant evidence that the regulation of inhibition is central for cortical plasticity throughout the lifetime. However, the ways through which the levels of cortical inhibition are controlled differ between the developing and mature brain. During the development, the cortex favors experience-driven recruitment of cortical inhibition through bottom-up thalamic pathways that relay sensory information (Cruikshank et al., 2007; Chittajallu and Isaac, 2010; Toyoizumi et al., 2013; Ribic et al., 2019). This way, learning through frontal brain areas that develop last in succession is bypassed (Kolb et al., 2012) and immediate environment may robustly shape sensory functions that are required early in life through passive exposure. After the frontal brain regions mature at the offset of adolescence, heightened inhibition and different structural molecular brakes on developmental plasticity, such as extracellular matrix (ECM) and myelin (Pizzorusso et al., 2002; McGee et al., 2005), restrict plasticity and it can no longer be robustly driven by simple exposure to stimuli. Initiation of plasticity instead plasticity requires modulation of cortical inhibition through attentional, top-down pathways from frontal cortical areas (Letzkus et al., 2011; Pinto et al., 2013). Plasticity can still occur through passive stimulation (Cooke and Bear, 2010), but attention enhances it, which may be a prerequisite for efficient learning in adults (Seitz and Dinse, 2007). In this way, adult circuits can maintain stability, while still being able to acquire new skills and adapt to novel contexts on a “need to know” basis. It is currently unclear whether developing and adult circuits are relying solely on bottom-up and top-down pathways, respectively, to regulate plasticity. Progress is hampered by lack of appropriate models of associative and passive learning in developing and adult experimental animals, respectively. Passive learning in adult animals has been reported (Cooke and Bear, 2010), but some forms also require cholinergic signaling (Gavornik and Bear, 2014; Kang et al., 2015). On the other hand, plasticity during vocal learning in juvenile birds may require inputs from the frontal areas, suggesting that top-down modulation can occur before the critical periods close (Puzerey et al., 2018). It would appear that developmental learning may not be as exclusively bottom-up as initially thought, and neuromodulatory tuning of inhibition may be a universal way for regulating circuit plasticity throughout the life span (Takesian et al., 2013; Yaeger et al., 2019). The development of new juvenile learning paradigms that explore more complex skills and behaviors may be central in identifying potential top-down pathways that may mediate learning during development (Bicks et al., 2020).

Pinpointing how and when plasticity occurs remains central to our understanding of learning and memory formation. Studies of developmental plasticity helped define key mechanisms that drive plasticity and learning in adults (Hubel and Wiesel, 1963, 1970; Wiesel and Hubel, 1963; Hubel et al., 1976; Frégnac et al., 1988, 1992). Inhibitory circuitry has proven key for plasticity in both developing and adult brains, and future studies will undoubtedly address ways to manipulate inhibition to enhance learning and recovery of function in different disorders. Now is the time to address whether attentional (neuro)modulation of plasticity typical for the adult brain can be used for learning during development. Distinct properties of plasticity in the juvenile brain may render some forms of learning, such as perceptual, not as efficient as in adults (Caras and Sanes, 2019). Understanding the mechanisms of skill acquisition through the engagement of frontal brain regions in youth will be invaluable in developing non-invasive therapies for recovery of sensory function in several neurodevelopmental disorders.

## Author Contributions

AR: drafting, figure design, editing, and corrections.

## Conflict of Interest

The author declares that the research was conducted in the absence of any commercial or financial relationships that could be construed as a potential conflict of interest.
